# Women and Insecurity in Nigeria: The Way Forward

**DOI:** 10.3389/fsoc.2022.734190

**Published:** 2022-07-07

**Authors:** Comfort Yemisi Afolabi

**Affiliations:** Centre for Gender and Development Studies, Ekiti State University, Ado Ekiti, Nigeria

**Keywords:** women, insecurity, insurgency, way forward, decision making, violence

## Introduction

The level of insecurity in Nigeria was very high for more than a decade. Attacks were carried out relentlessly by Fulani armed men, Boko Haram insurgents, bandits, and herdsmen in many towns and villages in Nigeria. The above scenario has led to the untimely death of over 456,831 innocent lives in Nigeria as of April 2019 (Duru, 2019; Ososanya, 2019; Sahara Reporters, 2019). The frequency and ferocity of these insurgencies in Nigeria were quite alarming and unprecedented in the annals of the country, and have unexpectedly led to insecurity on a large scale in Nigeria.

The major challenge that has been faced by the Nigerian government for more than 10 years is insecurity and this has led to the loss of lives and properties. The attacks took the form of the bombing of churches, schools, and police stations using improvised explosive devices (IEDs), shooting of innocent citizens mostly using AK47 rifles, abduction of school girls and women (including males sometimes), destruction of oil facilities, and the bombing of railway happened on the 28 March 2022 in Katari, Kaduna State. There were also instances of destruction of crops, large-scale burning of residential buildings, and kidnapping of passengers traveling on the road for ransom. As a result of the above security challenges, many citizens have been rendered homeless and many children have become orphans without any hope for the future. Apart from the above, national growth could be adversely affected by the issue of insecurity as exemplified above (Obi, 2015). Many girls kidnapped have been forced into marriage by members of Boko Haram. A good example was the case of Leah Sharibu who already had two children (BBC News, 2021). This situation was a demonstration of a high level of insecurity. According to Premium Times (2021), Nigeria was one of the most threatened and unsteady nations worldwide. Nigeria ranked third in the world with a death toll of 25,711 from 2010 to 2019, not including women and girls raped and captured by Boko Haram insurgents in Nigeria since 2020. The majority of Nigerians lived in perpetual fear and anxiety of attacks from criminal elements in the country as people were no longer safe in their homes. They were afraid to go to farms or embark on journeys. Apart from this, students dreaded going to school. In fact, many parents withdrew their children from schools in the northern part of the country. However, the burden was more on women and children because they were more hit by the clashes, insurrection, and violence. The The Global Right Atrocities Report (2021) said that 4,556 Nigerians were killed in 2020, especially in North-Eastern Nigeria. Furthermore, over 220 abductions and at least 2,114 fatalities were on record and over 600 schools were closed down in six Northern States in the first quarter of 2021. There were also violent attacks in Abia, Benue, Imo, Niger, Ondo, and Oyo States from non-state actors, traceable to Boko Haram insurgency and farmers–herders clash, among others.

According to the Joint Shadow Report by the Convention on the Elimination of All Forms of Discrimination against Women (CEDAW) Committee, 67th Session (2017), the quest by herdsmen to find sufficient grazing land for their cattle has resulted in violent farmers–herders clashes in some parts of the country like North-Eastern Nigeria, Niger Delta, North Central and Southeast Nigeria. As a matter of fact, in many rural communities where there were many female farmers, the fear of cattle destroying their crops has discouraged many of these female farmers from continuing their farming activities which have resulted in rising scarcity and poverty among many rural dwellers, especially women. What compounded the problem was that security agencies were not always around to protect these women farmers and men from savage and brutal attacks purposely carried out by herdsmen and other criminal elements.

These insecurity occurrences in Nigeria have resulted in the displacement of inhabitants of villages and towns torn by insurgency. Many people who were driven away from their homes by Boko Haram insurgents or killer herdsmen were kept in Internally Displaced Persons' Camps (IDPCs) that were established by the government to accommodate the displaced men, women, girls, and children. Internally displaced persons are people who were forced to leave their homes but remain within the border of their country. According to the Internally Displacement Monitoring Centre (2021), 169,000 inhabitants have been driven away from their homes due to violence from 1 January to 31 December 2020. The total number of IDPs that resulted from Boko Haram attacks and other conflicts as of 31 December 2020 was 2,730,000. The IOM Displacement Tracking Matrix noted that in Rounds 35 and 36, 2,150,243 and 2, 184, 254 persons were displaced from their homes in December 2020. These people were displaced from 447,628 households (IOM Displacement Tracking Matrix, 2021). More worrisome was the fact that women and girls in these IDP camps were sexually assaulted by males from within and outside the camps, such as other male IDPs and men whom the abused females regarded as their protectors in the camps. Akanbi et al. (2019) reported that IDPs experienced at least one form of violence, which may be sexual, physical, economic, and emotional wreaked by Boko Haram rebels.

Women in Nigeria have been playing significant roles in national security in spite of cultural, religious, and demographic challenges facing them. Women are the most affected by uncertainties and different types of insecurities in all the geographical regions of Nigeria including the Federal Capital Territory. However, they have been found to participate in suicide bombings and acted as informants to Boko Haram members and the killer herdsmen. Despite the fact that women were the most affected when it comes to the issue of security, they seemed overlooked compared to their male counterparts in decision-making when it comes to finding solutions to violence and physical insecurity in Nigeria. Nigeria was ranked 139 according to the World Economic Forum's Global Gender Gap Report in 2018 in terms of the gender gap in political enablement and decision-making. The Women Advocate Research and Documentation Centre (WARDC) and Nigerian Women Thrust Fund (NWTF) noted that the Nigerian Women Charter of Demand wanted 35% of women to be involved in all aspects of decision-making in all sectors of government (Oluyemi, 2016; Kelly, 2019 and Peace Direct, 2019). However, women have not been prioritized when it comes to making decisions on how to end insecurity in Nigeria. Women have the right to partake in decision-making, including that of insecurity in the country. The United Nations Development Programmes' (1994) Human Development Report showed that global security should be long-drawn-out to include seven areas, such as economic, health, community, food, environmental, human, and political security (Idike et al., 2020). Premium Times (2021) stated that there was a need for Nigeria to build on women's original awareness and involve them in the communal peace architecture as peacekeepers and mediators to end insecurity.

Feminist scholars and gender campaigners working in conflict zones have emphasized the need to concentrate on the roles played by men and women during clashes, the gender discrepancy effects of violent conflicts, the need to address the various challenges faced by women, and the need to increase their involvement in peace and security issues and decisions. In all aspects of government, including the military and policy approaches, feminists had worked to ensure that women were effectively represented (Savage, 2021). Insecurity issues in Nigeria appeared alarming and women's involvement in the decision-making hierarchy on insecurity seemed minimal. Consequently, this paper focused on the burning issues of insecurity in Nigeria and the extent to which women have been involved in decision-making on insecurity in the country. The study employed the descriptive research design using secondary data on insecurity-related issues.

## Statement of the Problem

There seemed to be incidents of insecurity in Nigeria but its level and extent are not known by many people. Also, women and children appeared to be the most adversely affected group by insecurity in the country but incidentally, they seemed less visible than their male counterparts when it comes to discussing issues related to how to tackle insecurity in the country. Furthermore, the issues of insecurity in Nigeria were solely reported in the newspapers meaning that researchers have not written much on this problem. Even the national dailies and medium through which this problem of insecurity has been discussed have failed to shed light on women's level of involvement in decision-making when stakeholders meet and take far-reaching decisions on how to solve or manage the problem of insecurity in Nigeria.

## Objectives of the Study

The objectives of the study were to:

- find out the level of incidents of insecurity in Nigeria;
- assess women's insecurity challenges in Nigeria;
- determine the level of involvement of women in decision-making on insecurity in Nigeria; and
- proffer solutions to the low level of involvement of women in decision-making when it comes to tackling insecurity in Nigeria.

## Research Questions

The study was guided by the following research questions.

- What was the level of incidents of insecurity in Nigeria?
- What were the insecurity challenges facing women in Nigeria?
- What was the level of involvement of women in decision-making on insecurity?
- How can the problem of low involvement of women in decision-making on how to tackle insecurity in Nigeria be solved?

## Significance of the Study

The study would provide stakeholders with necessary information on insecurity at the state and federal levels and on the level of insecurity in Nigeria. The study would also inform people about the need for women's involvement in tackling and managing issues of insecurity in Nigeria.

The study would acquaint people with the insecurity challenges faced by women in Nigeria and the hurdles that women and girls face in the IDPCs in the country. The study would enable the relevant stakeholders in the area of insecurity to be aware of the suffering of women in the IDPCs which hopefully would enable them to put a stop to the irregularities and abuse of women and girls in camps all over the country.

The study would enable state and federal governments to embark on laudable initiatives to involve women just like their male counterparts in the management of insecurity in Nigeria. The study would provide information for governments on the need to find means of bridging the recruitment/appointment gaps between men and women in top management positions in security architecture in Nigeria.

## Literature Review

For some years now, the insecurity and terrorism index in Nigeria rose from 6.95 in 2011 to 7.96 in 2012; 8.2 in 2013; 9.21 in 2014; 9.31 in 2015; 9.10 in 2016; 8.66 in 2017; 8.6 in 2018, and 8.31 in 2019. The index processed the direct impact of violence and anxiety on a scale from 0 (no impact) to 10 (highest impact) (Institute for Economics Peace, 2019). Notwithstanding a reduction compared to the preceding 5 years, Nigeria continued to be one of the countries with the uppermost insecurity threat levels in the world. In 2019, Nigeria documented the second largest number of deaths related to terrorism and insecurity worldwide (Trading Economics Strata, 2021). The actions of criminals using the banner of religion and herders turned into a serious cause of concern for Nigerians. This was connected to the long-time farmers–herders clash, but security experts thought that criminals and banished insurgents from other African nations may have broken into the ranks of the once peaceful herding community to perpetrate crimes and violence across Nigeria. The South-East region of the country is engulfed in the problem of the indigenous people of Biafra who are a separate group seeking autonomy from the Nigerian State (Omoniyi, 2021).

Continuous kidnapping of school children in Nigeria in the last 7 years seemed to be the new gold mine for criminals in recent times. This was especially a consequence of the huge cash that the government and individuals allegedly paid for the release of victims. As a result of the assaults on schools, over 618 schools were shut down by States in the North, thereby adding to the already deteriorating out-of-school students that Nigeria faced (Omoniyi, 2021). Diverse groups in Nigeria recourse to violence. The militant Islamic group, called Boko Haram, was devilishly active in Northern Nigeria. In the Southern part of Nigeria, the Niger Delta militants threatened war against the state (Trading Economics Strata, 2021). In some parts of Nigeria, ethno-religious crises, and farmers–herders clashes were common. According to Feminist Womanifesto Group (2021), insecurity jeopardized the continued survival of the country, and every citizen could be affected.

Insecurity is a state of lack of protection from danger in which one is not at ease with oneself. It is a situation that involves fear, anxiety, worry, and apprehension. Insecurity is the state of being open to danger or risk and the feeling of being unsafe (The NROC Project, 2021). There are various types of insecurity which include bodily insecurity, emotional insecurity, food insecurity, economic insecurity, political insecurity, and environmental insecurity among others but the focus of this paper was on physical insecurity which resulted from insurgency, terrorism, and other forms of violence. This type of insecurity involves killing and maiming people. Insecurity cuts people's life short and prevents the use of human potential, thereby disturbing access to long life (Stewart, 2004).

One of the major causes of the farmers–herders clash has to do with climate change which prompted the Fulani herdsmen to move from their settlements in the Northern parts of the country to other areas where they can graze their cattle. Climate change has led to desert encroachment, desertification, disappearance, or shrinking of Lake Chad, droughts, and other negative consequences affecting the Fulani cattle. This made the Fulani herdsmen, also known as Bororos, leave their communities and travel to Southern Nigeria where there were better vegetation, buoyant market chances, and hope. These herders were always in conflict with farmers for spaces to rear their animals. In fact, these herdsmen were moving down in their numbers, and they were confronting farmers working on their farms on a daily basis. The violence resulted in an unhealthy relationship between the Bororo Fulanis and Middle Belt farmers, Yoruba in the Southwestern States, and the Ibos in the Southern States; this is a serious issue of concern in Nigeria (Folami and Folami, 2013). Also, the uprisings in the South for the crude oil in the region added to the level of insecurity in the country. The religious conflicts between Christians and Muslims also powered insecurity in the nation. There were also cases of suicide bombing, border clashes between neighboring communities, ethno-religious crises, and so on which contributed to insecurity in Nigeria.

Due to insecurity issues, many Nigerians, especially in the North, have fled their homes and became IDPs. The cases of displacement in Nigeria were multilayered, complex, and overlapping. Boko Haram and other non-state armed groups (NSAGs) triggered significant displacement in the Northeast since 2012. Recurrent violence between Fulani pastoralists and Hausa farmers in Kastina, Sokoto, and Zanfara States of Nigeria became more rampant and rural banditry and criminal violence were on the rise (Internally Displacement Monitoring Centre, 2021). Criminal violence was also reported in Southern Nigeria, though data on displacement was rare. Clashes and violence led to increased new displacement in 2020 and about 2.7 million people were living in IDPCs at the end of 2020, there was an increase in the data for 2019. There were also secondary movements of IDPCs previously caused by violence, especially in the Northern part of the country. From 4 to 10 October 2021, 783 people were displaced in the Batsari Local Government Area of Katsina State (Internally Displacement Monitoring Centre, 2021). According to IOM Displacement Tracking Matrix (2021), 29,846 people were displaced from their homes in August 2021 from Benue, Borno, Kaduna, Katsina, Plateau, Sokoto, and Zamfara. Also, armed clashes displaced 573 in Isa Local Government Area of Sokoto State from 4 to 10 October 2021. Armed clashes between herdsmen, farmers, bandits, and local communities displaced 1,103 persons from 4 to 10 October 2021 in the Gumi Local Government Area of Benue State. Moreover, according to the United Nations High Commissioner for Refugees (UNHCR, 2021), a total number of 14,555 people were sent away from their homes in Benue, Borno, Katsina, and the Sokoto States in September 2021. All these displaced people were settled in IDPCs by the government. Over 17,000 babies have been lodged in Borno IDPCs since 2019 (Premium Times, 2021).

It is significant to note that IDPCs faced a lot of difficulties. One of them was that they were forced to flee or leave their homes. They were exposed to a number of dangers. Displaced persons suffered high rates of death than the general population in Nigeria. They were also abused bodily, financially, sexually, and emotionally at the camps. The Nigerian government bore the primary obligation for the shielding and wellbeing of IDPCs. If the national government was powerless or reluctant to meet their needs and tasks, the international communities have a role to play in endorsing efforts to ensure protection and to provide aid and solution to their problems (OCHA, 2021). According to Premium Times (2021), other challenges faced by internally displaced persons were food insecurity, unemployment, penury, lack of adequate health care, water, electricity, sanitation, sexual harassment, and abuse. Akanbi et al. (2019) and Women Media Centre (2021) agreed that instances of sexual abuse of women and girls in camps in Nigeria were very rampant and that the evil acts were committed by men whom they regarded as their protectors in the camps and other male IDPs. Some of the victims were raped which led to unwanted pregnancies and children for whom the women solely catered for. Inwlomhe (2021) reported that there were cases of sexual abuse including rape and maltreatment of women and girls in IDPCs in Adamawa, Borno, and Yobe. Government officials and other authorities in Nigeria raped and sexually abused women and girls displaced by the clashes with Boko Haram according to the report. The report added that the government neither did anything to defend the displaced women and girls nor ensured that they had access to adequate basic rights and services. Also, there was no serious punishment for the abusers, who were camp leaders, vigilante groups, policemen, and soldiers. In late July 2016, Human Rights Watch documented sexual abuse including rape and exploitation of 43 women and girls living in seven IDPCs in Maiduguri, the Borno State capital. There were security breaches in service delivery and access to justice for women and girls victims in the camps. It was also reported in an assessment in April 2016, that gender-based violence was a feature of displacement in the disasters of Northeast Nigeria. It also acknowledged the lack of prioritization of issues of gender-based violence programs designed by the humanitarian community in Nigeria as an issue. Moreover, Read (2017) also said that the culprits and perpetrators of gender-based violence (GBV) at IDPCs were civilians, military, and emergency management officers detailed to protect and support internally displaced persons.

Boko Haram, an Islamist terrorist group focused its attacks on government officials, Christians, school children, and Muslims who condemned their acts or were suspected of aiding the Federal Government of Nigeria. Boko Haram mainly attacked people in North-Eastern Nigeria. They cited increased western influence and corruption in the administration of the country as the reasons for their violent activities against people. Their action against Christians and schools was called jihad. The former leader of the group was Shekau. The terrorists have burnt churches, killed so many Christians praying in their Churches, and abducted mainly adult females/girls, some males, and many students (Human Rights Watch, 2012). Several students are still in their custody and the group was demanding millions of naira as ransom before they could be released to their parents.

The problem of insurgency initially started in Nigeria in the early 1990s over disagreements between foreign oil companies and a number of Niger Delta minority groups who felt that they were being exploited, particularly, the Ogoni and the Ijaw people. This continued until the government and the companies met some of their demands with the presidential amnesty program. However, there are some pockets of insurrection in the Niger Delta region. The Indigenous People of Biafra (IPOB) also contributed to cases of insecurity in Nigeria in their agitation for a Biafran nation. There was equally a non-violent demand by Yoruba in the Southwest for an Oduduwa State. The Middle Belt people also agitated for a separate nation. Though these two regions were non-violent, some of their protests had led to fear, injury, and death which are all parts of insecurity in the country.

The issue of internal security administration had attracted world attention since the events of the 11 September 2001 attacks on the World Trade Center in the United States. These attacks, together with similar attacks like the one in Istanbul in 2001 and the train bombing in Madrid, Spain in 2004 have made nations strengthen their internal security systems in order to meet the challenges of protecting citizens against terrorist activities (Alumona et al., 2019). According to Okolie-Osemene (2019), security is a vital prerequisite in the wherewithal of modern states and in the international political system. This was due to a result of the need for states to be recognized as really sovereign and to be able to preserve their territorial integrity without control by other states.

Of note is the fact that for a nation to achieve development and be successful in all areas, it is necessary to be gender friendly. With particular reference to the current topic, the point is that the female gender should not be seen as insignificant in the attempts of the Federal Government of Nigeria to solve the problem of insecurity in Nigeria because the process of security is governed by a principle called gender equality. The process of security is governed by a principle called gender equality. About 70% of the world's poorest were women. Nigerian women were an insignificant and weakened force (Idike et al., 2020). However, this development did not stop women from contributing to insecurity in Nigeria. According to Adams and Olajumoke (2016), women caused 26% of Boko Haram suicide bombing incidents in Northern Nigeria, 17% of oil bunkering in the Niger Delta region, 12% of the kidnapping in the Southern Nigeria, 38% of armed robbery in Southern Nigeria, 41% of cases of corruption, and 43% of political violence and uncertainty all over the country. The above statistics has clearly shown that the female gender has contributed to insecurity in Nigeria and that they cannot be ignored when thinking of how to stop the problem in the country.

In recent times in Nigeria, millions of students are becoming victims of the clashes caused by insecurity thereby leading to loss of lives and property. Security threats and challenges were acts or events that exposed the material or identity of individuals, societies, or states to dangers. National security threat contains programs of antagonistic governments, some resulting from foreign governments with hostile intentions. National security threat covers terrorism, insurrections, the proliferation of arms, cybercrime, natural calamities, and diseases (Idike et al., 2020). The national threat in Nigeria was due to some factors that included poor government policies, corruption, poverty, unemployment, and a weak judiciary system, among others. These have really affected the growth of the nation adversely (Idike et al., 2020). Obi (2015) found out that insecurity, such as terrorism had negative effects on the development of Nigeria. Insecurity has led to the diversion of resources meant for the development of the nation to procuring amenities, consulting security experts, recruiting additional soldiers, and seeking assistance from other nations through different diplomatic means.

## Theoretical Orientation

The theory underlying this study is the feminist theory that favored equal opportunity for all men and women in all societies and nations including women's equal involvement in issues of insecurity like men. The feminist theory initially emerged in 1794 through the publication of Mary Wollstonecraft, entitled “A Vindication of the Rights of Woman”; The Changing Woman, a Navajo Myth; and Ain't I a Woman in 1851 by Truth (2005) among others. The extension of feminism into theoretical, fictional, or philosophical dialogue can be called the feminism theory. Its objectives were to understand the nature of gender equality, examine the social roles of women and men, interests, responsibilities, and feminist politics in a variety of fields, such as anthropology, sociology, communication, media studies, psychoanalysis, ecology, home economics, literature, education, and philosophy (Brabeck and Brown, 1997). The themes covered by feminist theory often included discrimination, objectification, oppression, patriarchy, stereotyping, arts, history, and aesthetics (Lerman and Porter, 1990; de Zegher, 1999; Armstrong and de Zegher, 2006). The main forms of the feminist theory were Liberalism, Socialism/Marxism, and Radical Feminism (Cott, 1987). The main focus of all these feminist theories was enhancing gender equality in society and the inclusion of women in all programs, opportunities, chances, and choices like their male counterparts.

Feminism, simply put, is a range of movements and ideologies that shared the same goal which was to define, establish, and achieve equal political, economic, cultural, personal, and social rights for women. These encompassed the establishment of equal opportunities for women in education, decision-making in vital areas and opportunities (including decision-making on insecurity issues). Feminists pointed out that in most cultures throughout history, men have been given more opportunities than women (Curran and Renzetti, 1998). In essence, feminism aids women to have integrity and equality with men.

Regarding any serious academic discourse, people cannot discuss works on gender equality without reference to feminism. Feminism addressed the issues of gender inequality, patriarchy, and sexism. Feminism, therefore, advocated for a change toward greater equality between men and women, promoted the expression of opportunities and choices for women, favored the elimination of gender stratification and reproduction, and championed how to put an end to all forms of violence in and outside the home.

There existed a cultural dichotomy between the male and the female gender. Socially, women and men were not equal or treated the same way in the world. Women were exploited by men. Society was male-dominated and patriarchal. Feminism was borne out of this discriminatory situation. Feminism was a movement directed at turning things around and causing a change in the unnatural recognition, power, and honor given to the male gender. Feminism, therefore, believed that men and women should enjoy equal status socially, politically, and economically. This means that feminism promotes equality for both sexes. That is, women are valued as equals, have equal rights, chances, and capacities with men. The belief was that if women are given equal opportunities like men, they would contribute equally and significantly to the growth and development of any nation or society. Feminism is a recognition and analysis of male supremacy and the efforts to change it. Feminism solicited equal rights for men and women in any society (Curran and Renzetti, 1998).

The goals of feminism were to establish the importance of women in society and prove that women were not passive, reveal that women had been subservient to men, and as well bring about gender equality. Given these three goals, feminism was concerned with equality of men and women meaning that women should share with men equally the scarce national resources (including top positions in the army, police, and other security/insecurity architecture). It was a movement formed to bring about social, political, and cultural rights, especially for women. Nevertheless, it had become a contentious concept (for women, men, and religious groups among others) and it is often seen as a threat to men. Feminist theory is about the way societal norms, roles, values, and institutions have restricted women's behavior, choices, and opportunities, how gender inequality and gender oppression could be understood, and it also shows the way in which women's control can be explained.

## Methodology

The study employed the historical descriptive research design and used existing secondary data sources to access information. The secondary sources used included articles, dailies, journals, documents, books, and libraries on security issues.

## Incidents of Insecurity in Nigeria

The fact that various insurgent groups were attacking and killing army officers, the police, and other security agents is worrisome. These security agents were expected to overwhelm and eradicate the insurgencies in the country but the reality on the ground was the opposite which was frightening. Insecurity, such as terrorism and others were global issues that demanded urgent consideration from governments worldwide. In this direction, the Nigerian government had made desperate efforts to curtail insecurity challenges in the country, but the efforts had not yielded the desired result at all (Obi, 2015). These various forms of insecurity had overwhelmed the nation because of the huge number of fatalities recorded and the humongous expenses incurred in fighting it. Nevertheless, Olonisakin (2019) noted that the government's key concern was protecting the citizens from danger and fear. He added that the ability of any government to live up to its responsibility is hinged on its security architecture.

Note that the data that portray the level of incidents of insecurity in nigeria is uploaded separately as additional material.

The data are really alarming, outrageous, horrible, and saddening. Starting from 1980, kidnappings carried out for different reasons remained a feature of the landscape of criminal victimization in the country. Moreover, the menace of kidnapping for ransom (K4R) became a serious obstacle to human security in Nigeria (Onuoha and Okolie-Osemene, 2019). The new pattern of insecurity was the kidnapping of students from schools in Northern Nigeria by insurgent gangs who demanded millions of Naira for their release.

## Women and Insecurity Challenges in Nigeria

Women were not actively involved in insecurity issues, especially at the top or leadership level where efforts to put an end to the ills are made though they were the most adversely affected, counting their female children. According to Savage (2021), peace and security were areas in which women had particularly been relegated when key policy decisions were made, and resource distribution was decided. The continued relegation of women in peacebuilding and conflict determination processes had affected development, particularly at the local level since tradition did not inspire the leadership of women. According to Kolawole (2020), all over the world, women and children take the impact of the world's clashes and wars. During these clashes, they were often exposed to inconceivable dreadful mayhems like massacres, sexual assaults, kidnappings and slavery, forced marriage, disfigurements, and forced pregnancy. Rape is actually being used as a weapon during wars. It was a well-known fact that women and girls were largely invisible when it came to conflict management and peace building. It was not due to any biological or operational reasons that women or girls were unable to participate in conflict resolution. The point was that women were often seen only as feeble victims of violent wars, rather than as agents of change whose ability could be exploited in peace processes. However, the United Nations Security Council also recognized the importance of increasing women's involvement in conflict determination and peace-building processes, particularly at the decision-making level (O'Reilly et al., 2015). Adeni (2015) noted that there were some cultural obstacles affecting women when it comes to active participation in conflict resolution in their states.

An alliance of women groups under the aegis of Women's Voice and Leadership Nigerian Project, urged the Nigerian President, Muhammad Buhari, to engage more women in the decision-making process and in the management of the country including insecurity architecture. The body, which made the call at its second Annual National Women Conference held in Abuja, implored the President to raise the level of women's inclusion in the nation's workforce and leadership. The body went further to uphold that involving women in decision-making procedures would help the country to find solutions to some of the difficulties it was facing, especially, the issue of insecurity. The Director of Action Aid Nigeria opined that what people saw in Nigeria was conflicts fought over women's bodies. Women have been used as arms and targets of violence (Vanguard News, 2016). The uneven impact of insecurity on women, girls, and children could not be overlooked. The group revealed that Nigeria needed to act as fast as possible to stop this increasing insecurity in Nigeria. The Action Aid wanted Nigeria to operationalize in all regions of the country, the provisions of the United Nations Security Council Regulation 1325, which charged all states to enable women's involvement in leadership in the area of peace and security that Nigeria was committed to. Action Aid Nigeria called on all state and non-state actors to reconsider the country's security design and ensure women's presence in the peace processes so that insecurity would be eradicated (Vanguard News, 2016). Women could be engaged informally through peace contests, the conception of unions and organizing peace coalition. The country should understand that having women at the peace table does not mean that they would drive for a gender-sensitive approach in the process or would advocate for women's issues alone (Savage, 2021). According to Kolawole (2020), women's involvement in grassroots peace-building in Nigeria is very key to her development and growth.

The Joint Shadow Report of CEDAW Committee (2017) noted that women in Nigeria had paid a weighty price in the conflicts devastating the century, especially, in the past two decades. They have suffered unparalleled stages of sexual violence and internal dislocation. Constant clashes in Nigeria include those related to fierce fanaticism in the Northeast and clashes over resource control, kidnapping in the Niger Delta, and complaints over land use due to antagonism between farmers and herders. Conflicts in the Northeast have resulted in enormous loss of lives, properties, and areas of livelihood. Boko Haram assaults and increased militarization in the region continued to have overwhelmingly negative effects on women and girls. In addition to Boko Haram's abduction of girls, their recruitment as suicide bombers, sex slaves, and forced laborers, Boko Haram's attack had escalated the number of women and girls who had been internally banished and therefore forced to seek shelter in various camps across the Nigerian nation (Joint Shadow Report of CEDAW Committee, 2017).

Peace and security are areas in which women have been predominantly marginalized, especially where key policy resolutions are made, and allocation of resources are decided. Women's continual marginalization in peace-building and conflict-resolving procedures has affected the country's growth and development, predominantly at the local level, since custom does not inspire women's headship (Garba, 2016).

There should be a framework that addresses the root causes of conflict and at the same time, ensures that women's equal and meaningful involvement in peace-building and conflict-resolution processes and decision-making are fundamental elements of conflict avoidance. According to Onyejekwe (2009), the acknowledgment of the conflicts affecting women was well-recorded in conflict and peace-building works. Although women were severely affected by war through vulnerability to sexual and gender-based violence including rape, forced recruitment, sexual slavery, kidnapping, and forced impregnation among other ills, it does not mean that they cannot play more positive roles as well. To Hassan (2015), the issue of women not inhabiting top positions was a challenge in Nigeria and across all sectors because the majority of these positions (even in security outfits) are occupied mostly by men, therefore giving few chances to women.

There were challenges to women's involvement in decision-making in Nigeria. The National Action Plan aims to achieve one important thing for women which is making them relevant in peace and security issues in Nigeria and in the process, demands women's equal and full participation at all levels of decision-making including insecurity in the country (Premium Times, 2021). Womanifesto Group demanded that Nigeria should set up frameworks and mechanisms that will make it possible to detect potential conflicts very early and also ensure women's involvement in peace and security decision-making. The group added that Nigeria should launch an early warning and response mechanism to track, in real-time, when an incident occurred and when security agents responded. The group declared that incidents of invaders spending hours killing and injuring citizens in communities must end and that the country should build on women's indigenous information gathering methods and involve them in the community peace architecture, as intermediaries (Premium Times, 2021).

According to Quadri (2015), the more women asked for more presence in decision-making in the country through their various groups, the less their involvement in decision making. Hence, the efforts expended by Nigerian women on political agitations and appointment into management positions would be fruitless exercises in bureaucratic architecture and the democratic project of the country, if care is not taken to bring about the desired change. These fruitless agitations have not only negatively affected the development of the country but have also weakened the country's development. Savage (2021) opined that Women, Peace, and Security Programme was viewed by many as the most momentous global plan for increasing the role of women in conflict resolution. The risk to women and children and the importance of involving women in peace issues have been documented as a global concern. There was a growing understanding of violence against women, generally and particularly in armed conflicts including the issue of sexual violence. Women were often exempted from the peace process, thereby reproducing the fact that most of the actors in conflicts were men. There was a gap in the gender participation of women in issues relating to peace and security in Nigeria. Savage (2021) recommended that the Nigerian government should have legal structures and policies that would promote the presence of women in conflict resolutions.

## Women and Decision-Making on Insecurity in Nigeria

Decision-making is the process of making choices through the identification of a decision, assembling relevant information, and carefully evaluating other possible options (with a particular reference to insecurity issues in this case). Women are more likely to partake enthusiastically in both private and public decision-making if they have better information and economic power which in turn will boost their self-esteem and self-assurance under consideration. Various women's group demonstrated their effectiveness, authority, and influence in many establishments. Women are often vibrant leaders of transformation in that they stimulate women and men to get involved in asserting their rights, affecting their communities positively as well as safeguarding their planet or space. Their involvement in decision-making is necessary for good governance. When women are involved in decision-making, they could be very fruitful and successful, including in the area of insecurity in Nigeria.

Women appeared to be less involved and less visible in decision-making in the area of security or insecurity in Nigeria. Nigeria has involved several women in decision-making positions like Senators, Members of the House of Representatives, Chief Justice of Nigeria, Vice Chancellors; Deans of Faculties in Universities; Directors of Institutes; Heads of Departments in many Nigerian universities; Nigerian Ambassadors; Ministers of Finance, Aviation, Agriculture and Water Resources, Foreign Affairs, Transport; Mines and Steel Development, Petroleum Resources; Senior Special Assistants; Director General, NAFDAC; Nigeria's Permanent Representative to the United Nations' World Tourism Organisation and Deputy Secretary-General of the United Nations. However, women had not been made to head core agencies and other bodies in charge of insecurity in the country like the military and the police as they should. The only exceptions were the appointment of women like Mrs. Olusola Obada as Nigeria's Minister of State for Defense from 2011 to 2012 and then the substantive Minister of Defense from 2012 to 2013 (superintending over the Nigeria military structures) and Fidelia Njeze who was the Minister of State for Defense in July 2007 (Idike et al., 2020). It is worthy to note that even these women were merely Ministers of State for Defense and when Mrs. Olusola Obada became the substantive Minister of Defense, she only supervised the military structures. In other words, men were the heads of Ministers of the core agencies in charge of security/insecurity in Nigeria. The positions controlled by women in these security agencies were less important than those occupied by men which made men as more superior to women. Simply put, women were less visible in the leadership of defense ministries than in the other ministries. This factor accounted for the momentous gender imbalance in the Nigerian democratic structure, and it related more to the non-institutionalized male gender bias that resulted in masculine or male-dominated politics and bureaucratic appointment in the public civil service and security apparatus (Quadri, 2015; Orji et al., 2018). Men were the current power controllers in Nigeria. Understanding gender equality involved losing some of the powers enjoyed by men to women (Idike et al., 2020).

There were no women appointed directly as the Minister of Defense and in other security-related institutions. Also, women in top positions in the military were never appointed as the Chief of Army Staff; Chief of Defense Staff; Chief of Naval Staff, and Air Marshal. Also, there have been no women Inspector General of Police or Controller General of Immigration or Commandant General of Nigeria Security and Civil Defense Corps, and Director General of the State Security Service (SSS) among others. Adams and Olajumoke (2016) noted that only 30% of women were in the military service as against 70% who were men. This is an indication that the involvement of women in decision-making on insecurity issues in Nigeria was negligible. According to Idike et al. (2020), national development continued to be stunted in Nigeria as gender-balanced representation remained compromised (including the headship of security agencies). According to Pereira (2009), Chinekezi (2014), Okemakinde (2014), and Frazier (2016), African and Nigerian women had a secondary role to play than their male counterparts. There were twice as many females below the poverty line as males and up to 19 times more men than women were in decision-making posts including leadership and decision making on combating insecurity in Nigeria.

The military and other security agents were charged with protecting the Federal Republic of Nigeria, encouraging Nigeria's global security concerns, and supporting peacekeeping efforts, especially in Africa. This was a result of the doctrine called Pax Nigeriana. The Nigerian military consisted of the army, navy, and air force. The military in the country had played important role in the country's history since independence. According to Olonisakin (2019), most nations of the world have attained peace as the main landmark for their people, stating that the pursuit of peace was often combined with the quest for security. He added that all the security operatives were devoted to ending terrorism and banditry in Nigeria. Being Africa's most populated nation, Nigeria had repositioned its military as a peacekeeping force on the continent since 1995. The Nigerian military, through ECOMOG instructions, had been deployed as peacekeepers to Liberia in 1997; Ivory Coast from 1997 to 1999, and Sierra Leone from 1997 to 1999. With the mandate of the African Union, Nigeria stationed her forces in Sudan's Darfur region in order to bring peace back there. The Nigerian army had also been deployed across West African countries to combat terrorism in nations, such as Senegal, Chad, and Cameroon, as well as dealing with the Mali War including getting Yahya Jammeh out of power in 2017 (O'Loughlin, 1998; Lancia, 2011). It is important to note that women did not hold any significant leadership posts in all of the peacekeeping efforts mentioned above. The leadership of all ECOMOG teams was mostly men.

The girl-child continued to be under-represented in governments, legislative bodies, and in many other crucial sectors affecting public opinion, such as mass media, the arts, religion, culture, and insecurity in Nigeria. Worldwide, there were only 16% of countries where more than 15% of ministerial positions were held by women; however, in 59 countries, there were no female ministers at all (UN Women, 2021). Although women had the right to vote in nearly every country globally, there were few females in government. In 1994, only 10% of the world's Parliamentary Deputies were females (FAO, 1995). As of 1 April 2019, the global average of women in assemblies was 24.3% and as of October 2019, the global involvement rate of females in the national-level parliament was 24.5% (Inter-Parliament Union, 2020). Females accounted for 8% of all national leaders and 2% of all presidential posts in 2013 (Jalaza, 2016). These global leadership representations depict a picture of gender disparity against women as it happens even in the sphere of insecurity.

Even at the IDPCs in Northern Nigeria where displaced people fled, women were not involved in the headship of decision-making and at the management level like men. Some of the men leaders in the camps were found to be sexually abusing the female IDPCs. Women leaders may not do this if they are made to head the camps. There were very few females in Nigeria's political arena both at the state and federal levels, that is headship of Nigerian establishments saddled with political responsibilities (and insecurity in the country) (Akudo, 2013).

The obstacles faced by women's participation in politics, including security, were negative attitudes toward women in leadership positions. Women continued to suffer from election violence, intimidation, or hate speeches, and this discouraged women's participation. In order to increase the number of women working in government agencies (including security structures), the NWTF utilized networking chances, funding, mentoring, and training for leadership and advocacy (Oluyemi, 2016; Kelly, 2019; Peace Direct, 2019). Aluko (2011) discovered that political leadership (and headship of security operatives) in Nigeria was stratified on the bases of gender distinction, thereby questioning the adoption of gender neutrality in the political and appointment process of the nation (including decision making) by the stakeholders in the area of security.

The gender disparity in the nation's politics and appointments, including the security sector, was rooted in cultural history, characteristic of patriarchal African societies (Izueke and Ezichi-Ituma, 2018). Gender inequality in the country was enhanced by different cultures and beliefs which subordinated women to men. In most parts of Nigeria, women were considered subordinates to their male counterparts, especially in the Northern States and in other areas (Babalola, 2014; Mp3bullet, 2021) including the gender gap against women in the headship of the army and other security agents who were in charge of security in Nigeria.

According to Kolawole (2020), evidence showed that peace-building and resolution processes had higher proportions of success and were more likely to last when women were significantly involved. An analysis of 40 peace processes in 25 countries over three decades revealed that an agreement would always be reached when women's groups were able to effectively affect a peace process. From just being the victims of the harm caused by war, women in the 25 countries referred to the above-assumed leadership roles and in the process were able to address the causes, and consequences of the lingering crises unlike what obtained in Nigeria where women did not play any leadership or significant role in conflict resolution and management. The involvement of Nigerian women in peace-building has been a very marginalized and unbalanced one since the rise of violent clashes in Nigeria. Nigerian women had taken peace-building initiatives only within the non-formal sphere at the grassroots or community level. This was because it happened to be the only medium available to them through avenues, such as non-governmental civil society groups, informal female-based groups, and very little or no involvement at the governmental level.

## Conclusion

The study examined the level of insecurity in Nigeria. The study specifically addressed security challenges facing women in the country and their level of involvement in decision-making processes toward putting an end to the security challenges. Apart from the above, the study proffered solutions for ending the security challenges in the country. Through the statistics provided, the study was able to prove that there was a high level of insecurity in Nigeria. This situation of insecurity has led to the death of thousands of people through insurgency, kidnapping, banditry, and armed robbery in the country.

The statistics provided indicated the number of people killed and injured as well as the locations, dates, and perpetrators of the acts of insecurity. The causes of all these alarming massacres were many, such as religious conflicts between Christians and Muslims, revenge for the January 15, 1966 coup when many Northern leaders were killed by the Igbos, the Nigeria-Biafra war, internal conflicts, Ogoni crises on environmental degradation and pollutions by crude oil refineries in Balyesa State, Cross Rivers State, and Rivers State, the conflict with soldiers at Odi, various attacks by Boko Haram insurgents, and the struggle between farmers and the Fulani herdsmen over land. Apart from the above, there is the issue of kidnapping for ransom. In all the above, women and children were the worst affected by insecurity in Nigeria. Women bore a lot of burden during insecurity. However, women were less visible than men when talking of the headship of the army and other security agencies in charge of the administration of insecurity. All the number one officers in the army, police, and other security agencies had always been men.

## The Way Forward

Women and girls in Nigeria have basic human rights to be enlightened on; they should be given equal standards and quality education in order to be well developed to enable them to occupy their places in the employment spheres including the officer cadres in the army, police, and other security agencies. They should also be able to function as leaders in IDPCs. Female education could have an important effect on the development of a nation, and this could lead to empowered, productive, and active citizens. Adequate education of the girl-child develops growth rates and reduces social disparities. Females with higher educational qualifications would function in formal wage employment than those with elementary education. Education gives women chances and choices for a lifelong acquisition of knowledge, values, attitudes, skills, technological abilities, competence, and talents to compete with men for opportunities and allocation of available resources. More women and girls should be adequately educated like men and boys.

Women should be appointed or promoted to the post of substantive Minister of Defense, Chief of Army Staff, Chief of Defense Staff, Chief of Naval Staff, Air Marshal, Inspector General of Police, Comptroller General of Immigration, Commandant General of Nigeria Security and Civil Defense Corps, Director General of the State Security Service (SSS), and heads of IDPC. In other words, women should be appointed, like men to top posts/ranks in the Nigerian security architecture and establishments in order to enhance their efficiency and effectiveness in their operations against cases of insecurity in the country.

The appointments of heads of the army, police, and other security agencies should be done with a gender-sensitive lens to enable women to display their potential and capacity in resolving issues of insecurity in Nigeria. Gender equality is an integral component of every aspect of the social, economic, daily, official, and private lives of individuals and society with reference to men and women. There is a need to mainstream gender equality into all appointments into decision-making positions in the army and other security agencies saddled with tackling security/insecurity issues in Nigeria. Gender Mainstreaming means ensuring that women and men have equal access to and control over resources, development benefits, appointments, recruitments, and decision-making at all stages of development processes, projects, programs, or policies. Gender equality is the ultimate goal of gender mainstreaming.

Government should safeguard women's involvement in peace and security resolution-making as well as build on women's native knowledge for the purpose of including them in community construction as peacekeepers, envoys, and negotiators.

Since insecurity was the major challenge in Nigeria, the government at the federal, state, and local levels should do all it could to protect the nation from internal and external aggressions. Emergency aid, conflict avoidance and resolution, and peace-building should be the main concerns of Nigeria at present.

Government should seek help and advice from countries that have tackled insecurity challenges and have overcome them to a reasonable extent. The Nigerian Military should be empowered more with the required arms to fight insurgency. Adequate ammunition, vehicles, and other required equipment should be acquired for all security agents to enable them to tackle the rebels headlong and put an end to insecurity in the country or reduce it to the barest minimum. Government should improve security surveillance in the Northern, Eastern, and Southern parts of the country to curb the danger of insecurity. A provision could be made for grazing land for cattle.

Government should bridge the gaps of security intellect that existed between the ordinary citizens and the various security agencies in Nigeria, provide information and communication technological (ICT) innovation that would be used to detect any form of security threats, and make available security gadgets that would be used to neutralize explosive devices. All these, if put in place, could end incidences of insecurity in the country.

## Author Contributions

The author confirms being the sole contributor of this work and has approved it for publication.

## Conflict of Interest

The author declares that the research was conducted in the absence of any commercial or financial relationships that could be construed as a potential conflict of interest.

## Publisher's Note

All claims expressed in this article are solely those of the authors and do not necessarily represent those of their affiliated organizations, or those of the publisher, the editors and the reviewers. Any product that may be evaluated in this article, or claim that may be made by its manufacturer, is not guaranteed or endorsed by the publisher.
