# Supplementary material for: Women and Insecurity in Nigeria: The Way Forward
Source: Front Sociol. 2022 Jul 7;7:734190. doi: 10.3389/fsoc.2022.734190 (PMC9301453; doi:10.3389/fsoc.2022.734190)
Supplement: Supplementary file 1 [file Presentation_1.PPTX]

## Slide 1
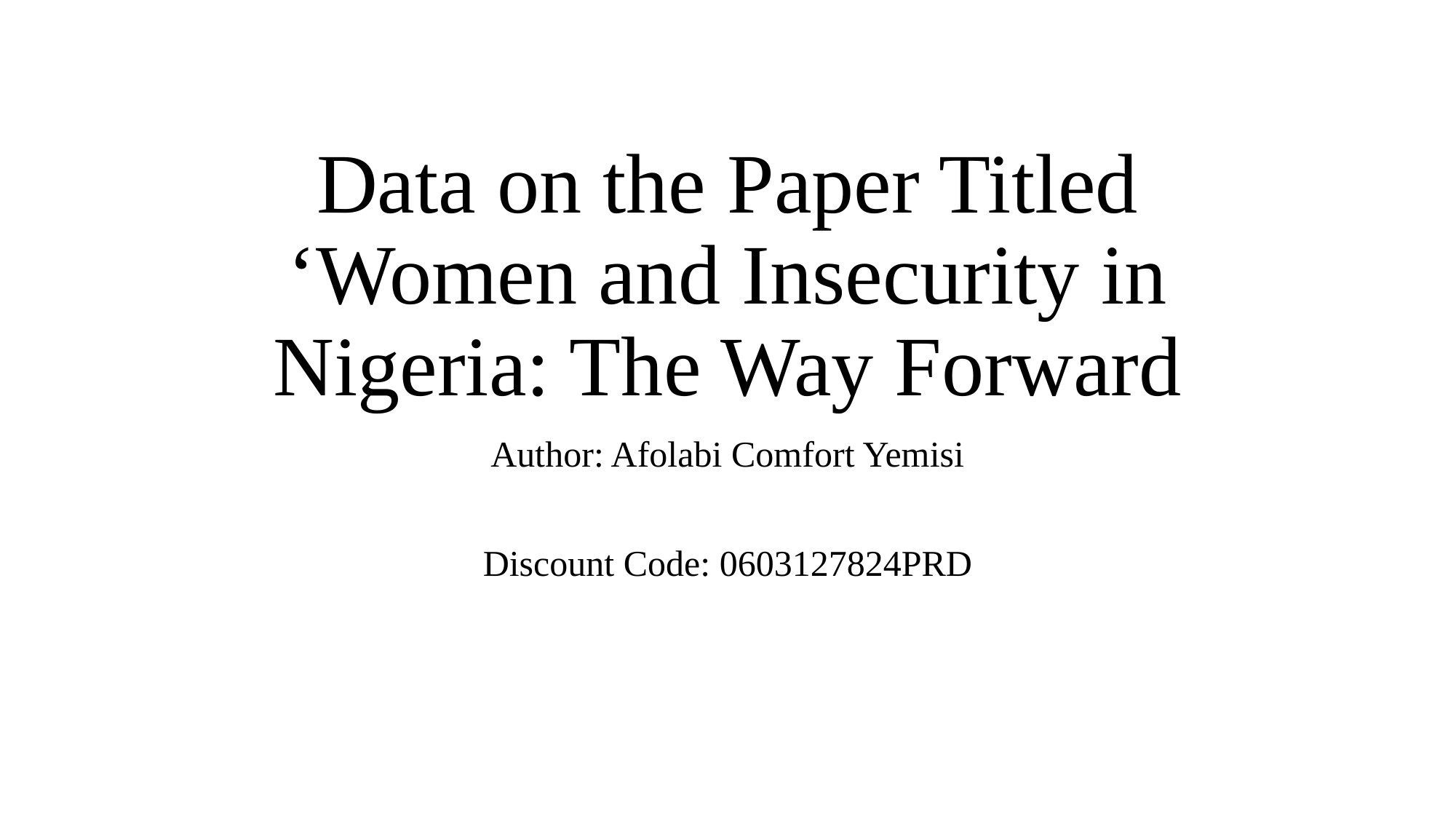

# Data on the Paper Titled ‘Women and Insecurity in Nigeria: The Way Forward
Author: Afolabi Comfort Yemisi
Discount Code: 0603127824PRD

## Slide 2
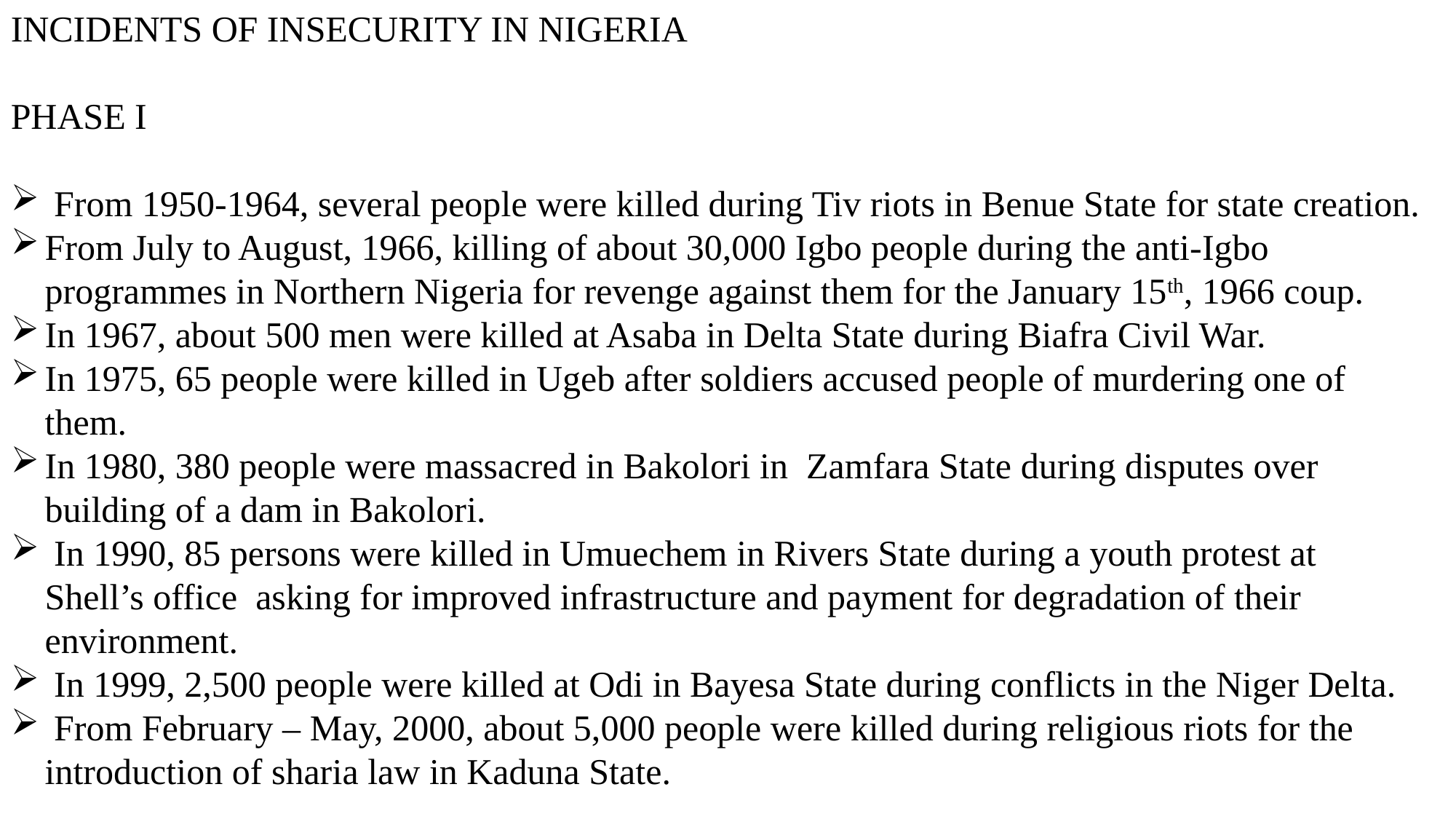

INCIDENTS OF INSECURITY IN NIGERIA
PHASE I
 From 1950-1964, several people were killed during Tiv riots in Benue State for state creation.
From July to August, 1966, killing of about 30,000 Igbo people during the anti-Igbo programmes in Northern Nigeria for revenge against them for the January 15th, 1966 coup.
In 1967, about 500 men were killed at Asaba in Delta State during Biafra Civil War.
In 1975, 65 people were killed in Ugeb after soldiers accused people of murdering one of them.
In 1980, 380 people were massacred in Bakolori in Zamfara State during disputes over building of a dam in Bakolori.
 In 1990, 85 persons were killed in Umuechem in Rivers State during a youth protest at Shell’s office asking for improved infrastructure and payment for degradation of their environment.
 In 1999, 2,500 people were killed at Odi in Bayesa State during conflicts in the Niger Delta.
 From February – May, 2000, about 5,000 people were killed during religious riots for the introduction of sharia law in Kaduna State.

## Slide 3
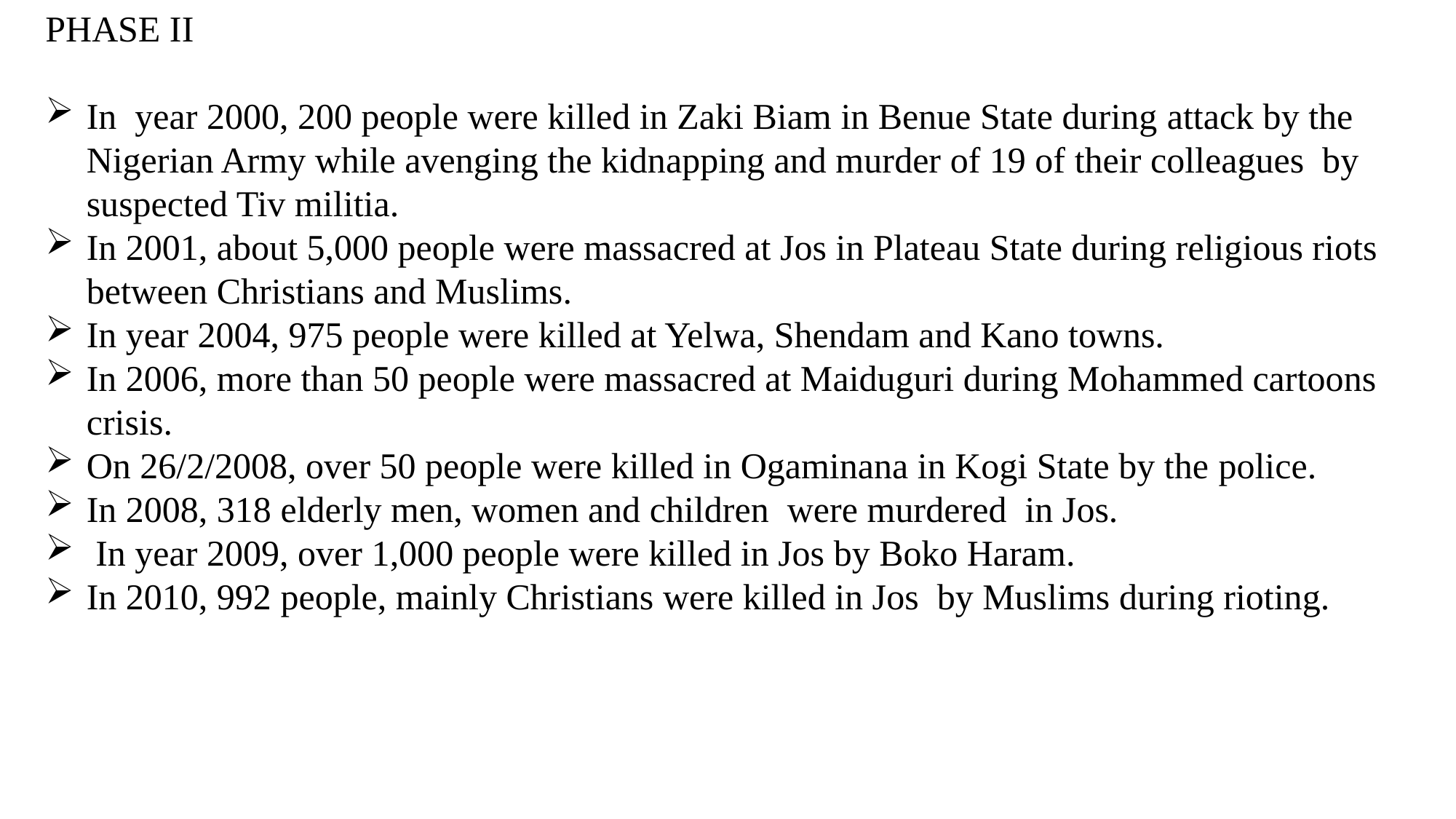

PHASE II
In year 2000, 200 people were killed in Zaki Biam in Benue State during attack by the Nigerian Army while avenging the kidnapping and murder of 19 of their colleagues by suspected Tiv militia.
In 2001, about 5,000 people were massacred at Jos in Plateau State during religious riots between Christians and Muslims.
In year 2004, 975 people were killed at Yelwa, Shendam and Kano towns.
In 2006, more than 50 people were massacred at Maiduguri during Mohammed cartoons crisis.
On 26/2/2008, over 50 people were killed in Ogaminana in Kogi State by the police.
In 2008, 318 elderly men, women and children were murdered in Jos.
 In year 2009, over 1,000 people were killed in Jos by Boko Haram.
In 2010, 992 people, mainly Christians were killed in Jos by Muslims during rioting.

## Slide 4
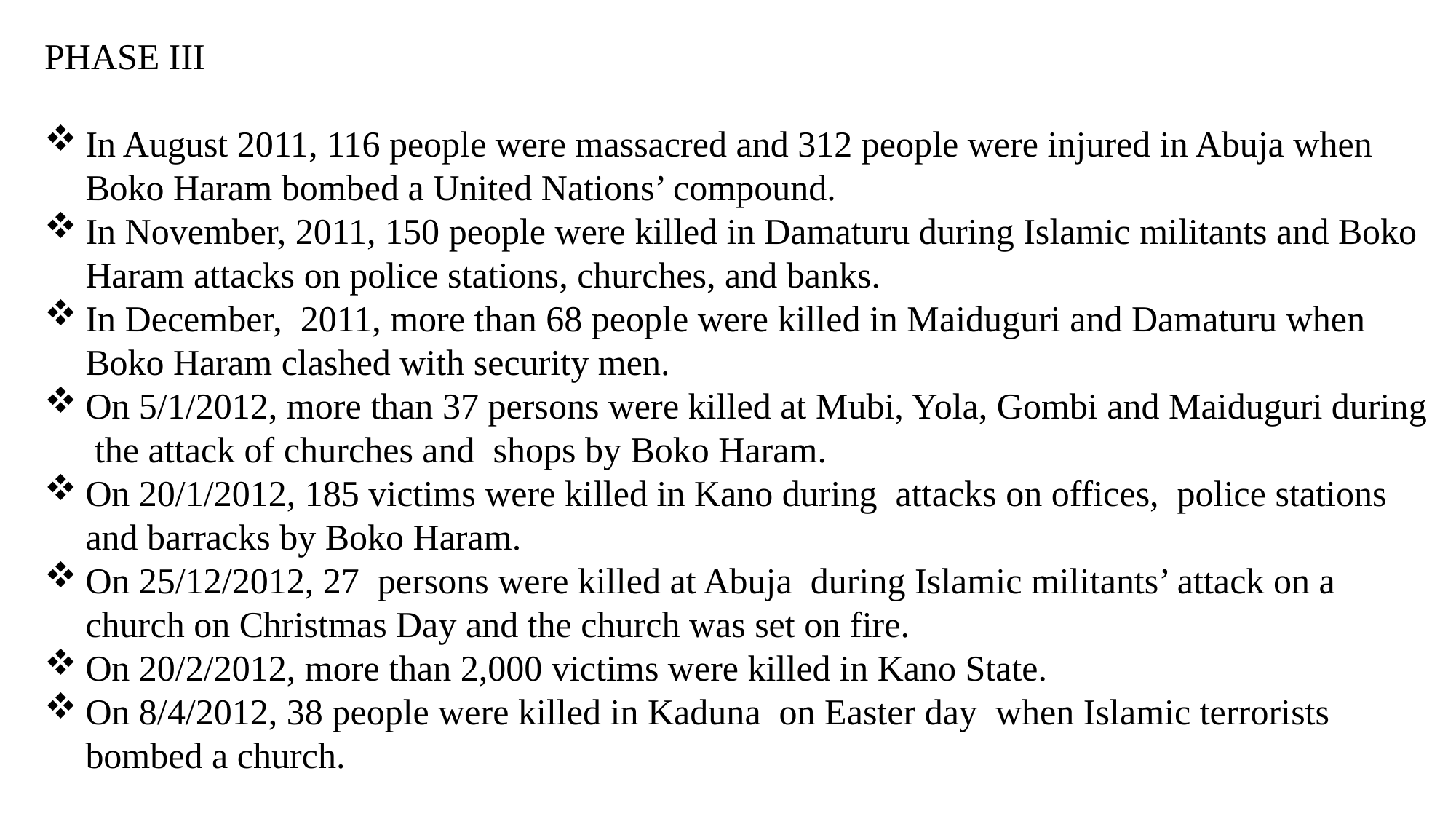

PHASE III
In August 2011, 116 people were massacred and 312 people were injured in Abuja when Boko Haram bombed a United Nations’ compound.
In November, 2011, 150 people were killed in Damaturu during Islamic militants and Boko Haram attacks on police stations, churches, and banks.
In December, 2011, more than 68 people were killed in Maiduguri and Damaturu when Boko Haram clashed with security men.
On 5/1/2012, more than 37 persons were killed at Mubi, Yola, Gombi and Maiduguri during the attack of churches and shops by Boko Haram.
On 20/1/2012, 185 victims were killed in Kano during attacks on offices, police stations and barracks by Boko Haram.
On 25/12/2012, 27 persons were killed at Abuja during Islamic militants’ attack on a church on Christmas Day and the church was set on fire.
On 20/2/2012, more than 2,000 victims were killed in Kano State.
On 8/4/2012, 38 people were killed in Kaduna on Easter day when Islamic terrorists bombed a church.

## Slide 5
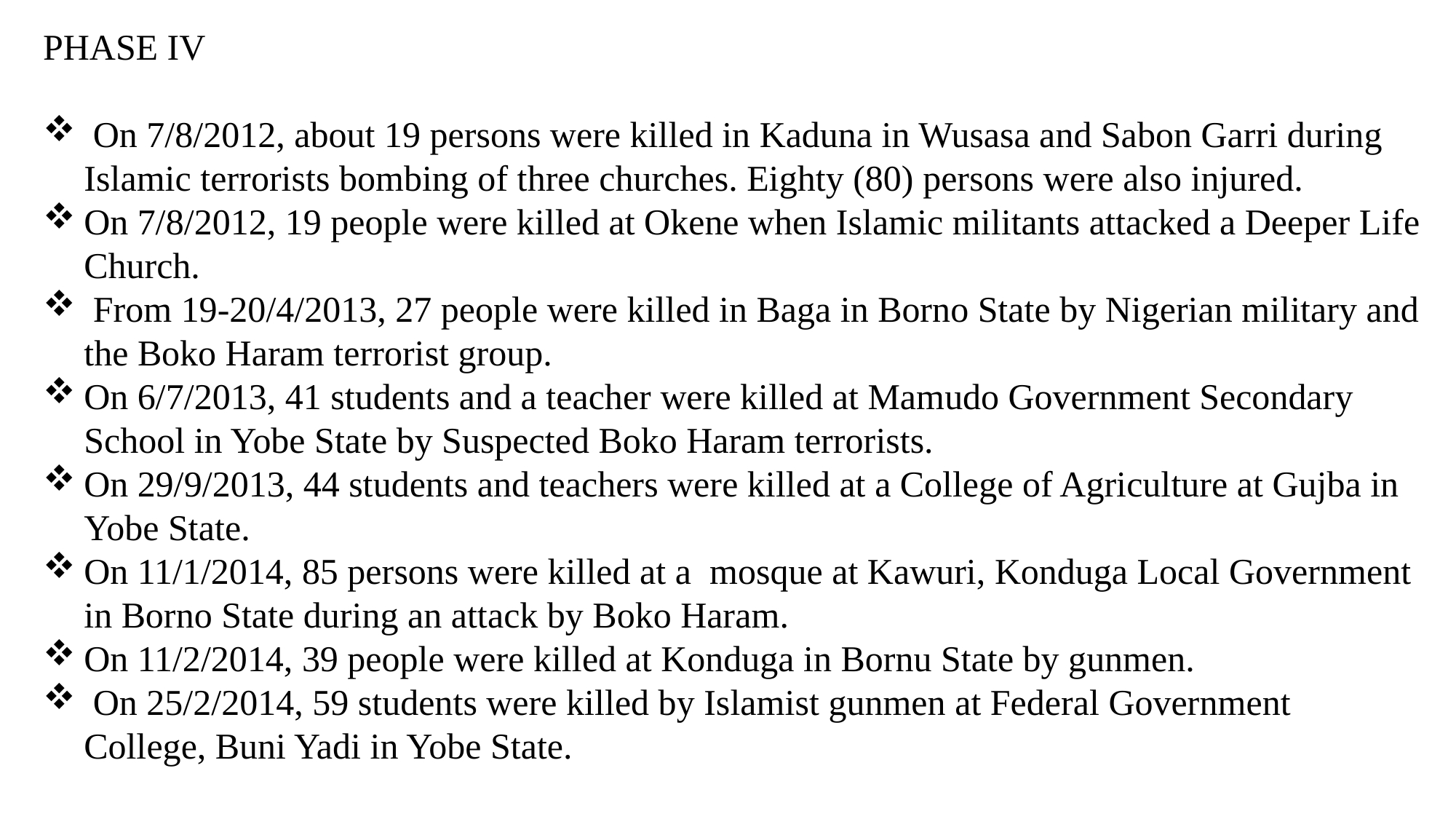

PHASE IV
 On 7/8/2012, about 19 persons were killed in Kaduna in Wusasa and Sabon Garri during Islamic terrorists bombing of three churches. Eighty (80) persons were also injured.
On 7/8/2012, 19 people were killed at Okene when Islamic militants attacked a Deeper Life Church.
 From 19-20/4/2013, 27 people were killed in Baga in Borno State by Nigerian military and the Boko Haram terrorist group.
On 6/7/2013, 41 students and a teacher were killed at Mamudo Government Secondary School in Yobe State by Suspected Boko Haram terrorists.
On 29/9/2013, 44 students and teachers were killed at a College of Agriculture at Gujba in Yobe State.
On 11/1/2014, 85 persons were killed at a mosque at Kawuri, Konduga Local Government in Borno State during an attack by Boko Haram.
On 11/2/2014, 39 people were killed at Konduga in Bornu State by gunmen.
 On 25/2/2014, 59 students were killed by Islamist gunmen at Federal Government College, Buni Yadi in Yobe State.

## Slide 6
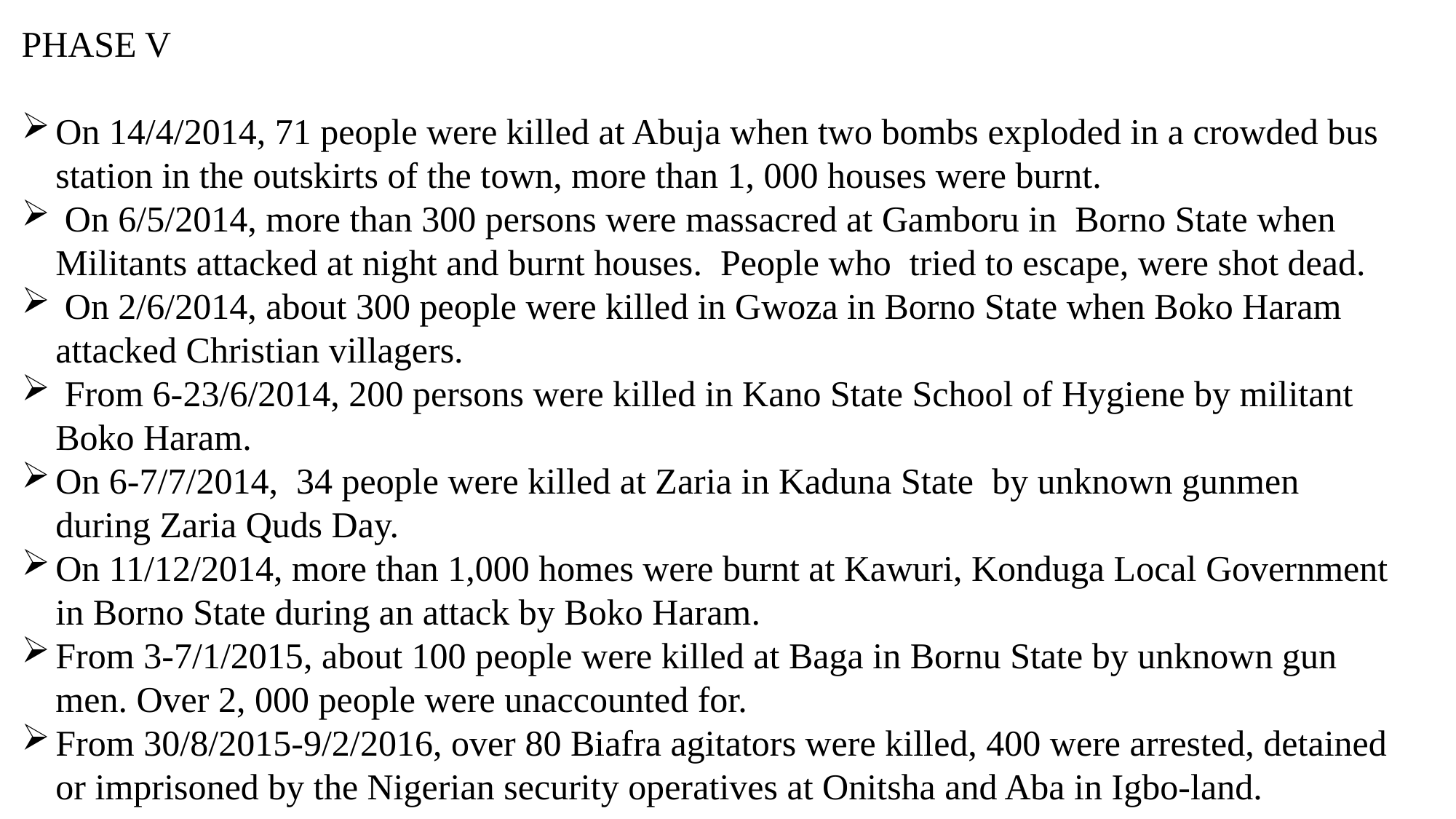

PHASE V
On 14/4/2014, 71 people were killed at Abuja when two bombs exploded in a crowded bus station in the outskirts of the town, more than 1, 000 houses were burnt.
 On 6/5/2014, more than 300 persons were massacred at Gamboru in Borno State when Militants attacked at night and burnt houses. People who tried to escape, were shot dead.
 On 2/6/2014, about 300 people were killed in Gwoza in Borno State when Boko Haram attacked Christian villagers.
 From 6-23/6/2014, 200 persons were killed in Kano State School of Hygiene by militant Boko Haram.
On 6-7/7/2014, 34 people were killed at Zaria in Kaduna State by unknown gunmen during Zaria Quds Day.
On 11/12/2014, more than 1,000 homes were burnt at Kawuri, Konduga Local Government in Borno State during an attack by Boko Haram.
From 3-7/1/2015, about 100 people were killed at Baga in Bornu State by unknown gun men. Over 2, 000 people were unaccounted for.
From 30/8/2015-9/2/2016, over 80 Biafra agitators were killed, 400 were arrested, detained or imprisoned by the Nigerian security operatives at Onitsha and Aba in Igbo-land.

## Slide 7
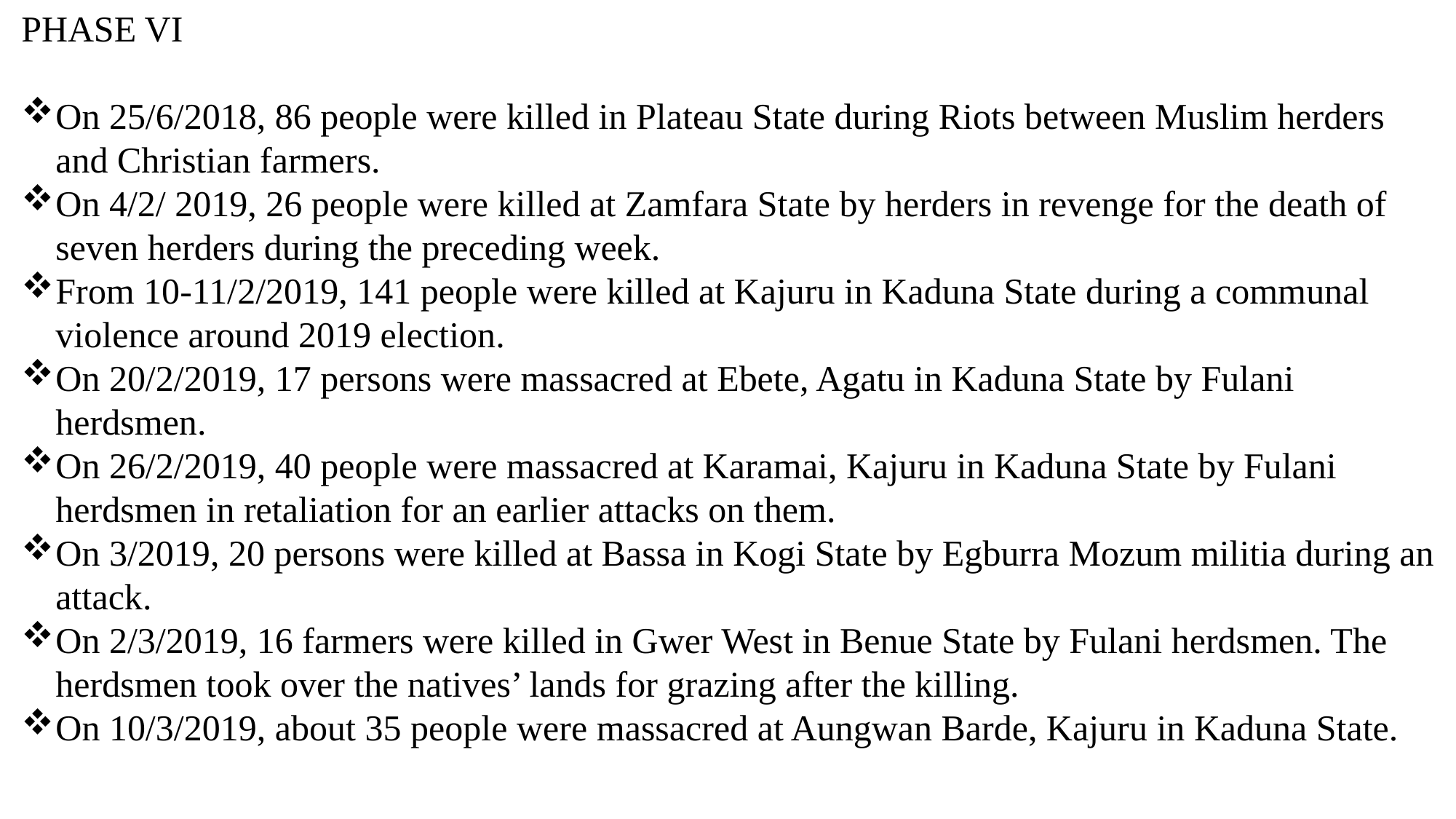

PHASE VI
On 25/6/2018, 86 people were killed in Plateau State during Riots between Muslim herders and Christian farmers.
On 4/2/ 2019, 26 people were killed at Zamfara State by herders in revenge for the death of seven herders during the preceding week.
From 10-11/2/2019, 141 people were killed at Kajuru in Kaduna State during a communal violence around 2019 election.
On 20/2/2019, 17 persons were massacred at Ebete, Agatu in Kaduna State by Fulani herdsmen.
On 26/2/2019, 40 people were massacred at Karamai, Kajuru in Kaduna State by Fulani herdsmen in retaliation for an earlier attacks on them.
On 3/2019, 20 persons were killed at Bassa in Kogi State by Egburra Mozum militia during an attack.
On 2/3/2019, 16 farmers were killed in Gwer West in Benue State by Fulani herdsmen. The herdsmen took over the natives’ lands for grazing after the killing.
On 10/3/2019, about 35 people were massacred at Aungwan Barde, Kajuru in Kaduna State.

## Slide 8
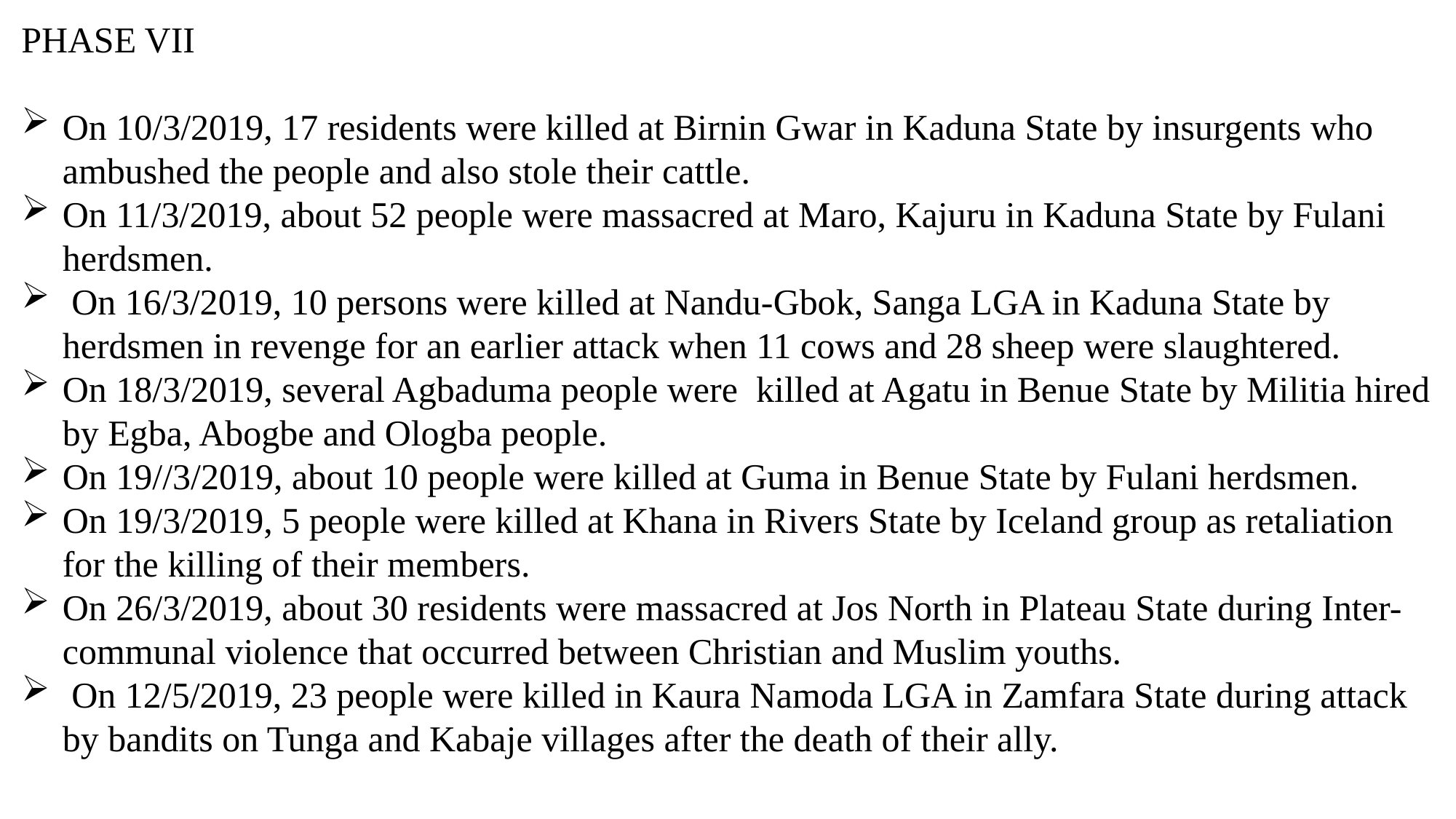

PHASE VII
On 10/3/2019, 17 residents were killed at Birnin Gwar in Kaduna State by insurgents who ambushed the people and also stole their cattle.
On 11/3/2019, about 52 people were massacred at Maro, Kajuru in Kaduna State by Fulani herdsmen.
 On 16/3/2019, 10 persons were killed at Nandu-Gbok, Sanga LGA in Kaduna State by herdsmen in revenge for an earlier attack when 11 cows and 28 sheep were slaughtered.
On 18/3/2019, several Agbaduma people were killed at Agatu in Benue State by Militia hired by Egba, Abogbe and Ologba people.
On 19//3/2019, about 10 people were killed at Guma in Benue State by Fulani herdsmen.
On 19/3/2019, 5 people were killed at Khana in Rivers State by Iceland group as retaliation for the killing of their members.
On 26/3/2019, about 30 residents were massacred at Jos North in Plateau State during Inter-communal violence that occurred between Christian and Muslim youths.
 On 12/5/2019, 23 people were killed in Kaura Namoda LGA in Zamfara State during attack by bandits on Tunga and Kabaje villages after the death of their ally.

## Slide 9
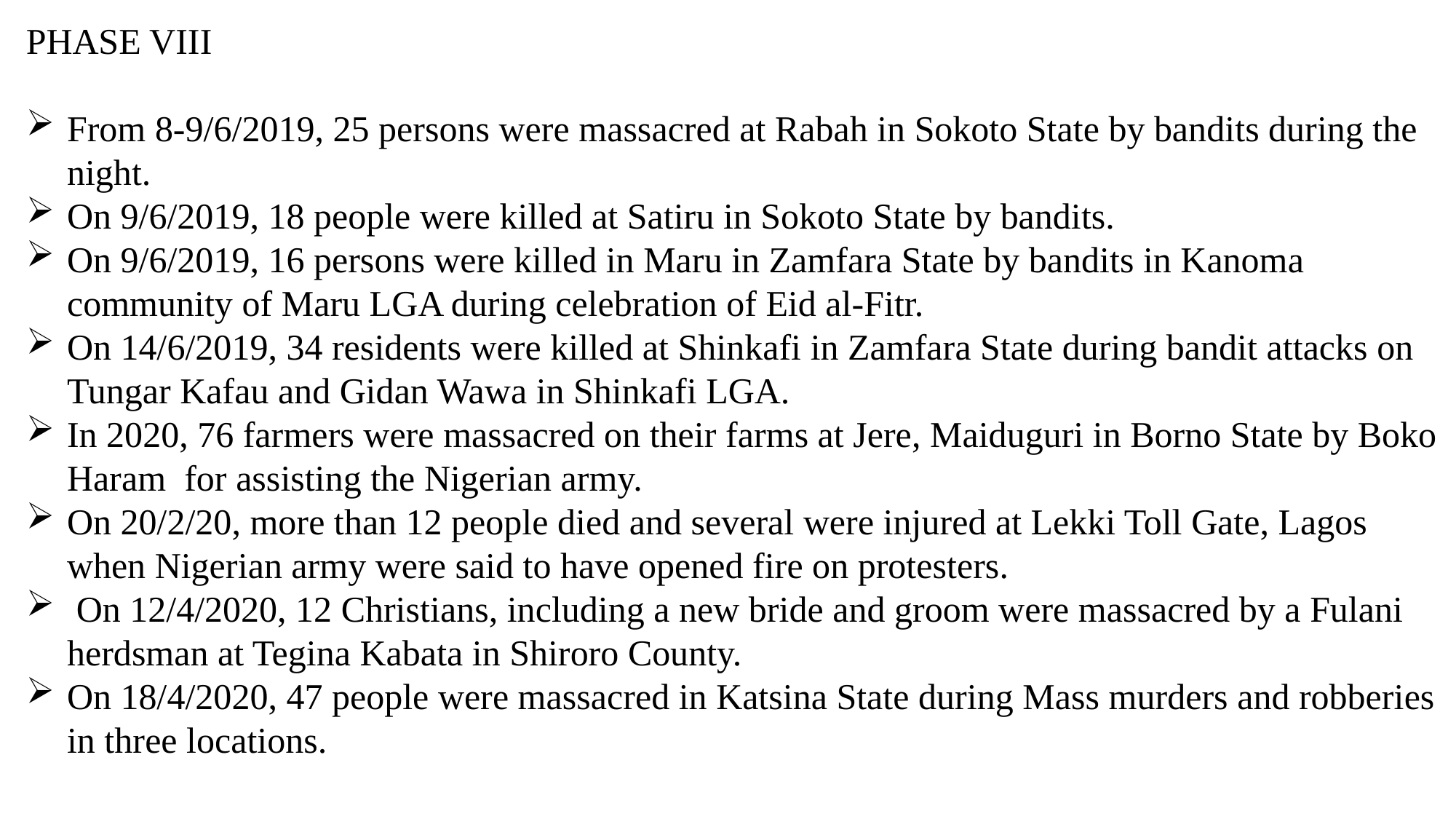

PHASE VIII
From 8-9/6/2019, 25 persons were massacred at Rabah in Sokoto State by bandits during the night.
On 9/6/2019, 18 people were killed at Satiru in Sokoto State by bandits.
On 9/6/2019, 16 persons were killed in Maru in Zamfara State by bandits in Kanoma community of Maru LGA during celebration of Eid al-Fitr.
On 14/6/2019, 34 residents were killed at Shinkafi in Zamfara State during bandit attacks on Tungar Kafau and Gidan Wawa in Shinkafi LGA.
In 2020, 76 farmers were massacred on their farms at Jere, Maiduguri in Borno State by Boko Haram for assisting the Nigerian army.
On 20/2/20, more than 12 people died and several were injured at Lekki Toll Gate, Lagos when Nigerian army were said to have opened fire on protesters.
 On 12/4/2020, 12 Christians, including a new bride and groom were massacred by a Fulani herdsman at Tegina Kabata in Shiroro County.
On 18/4/2020, 47 people were massacred in Katsina State during Mass murders and robberies in three locations.
.

## Slide 10
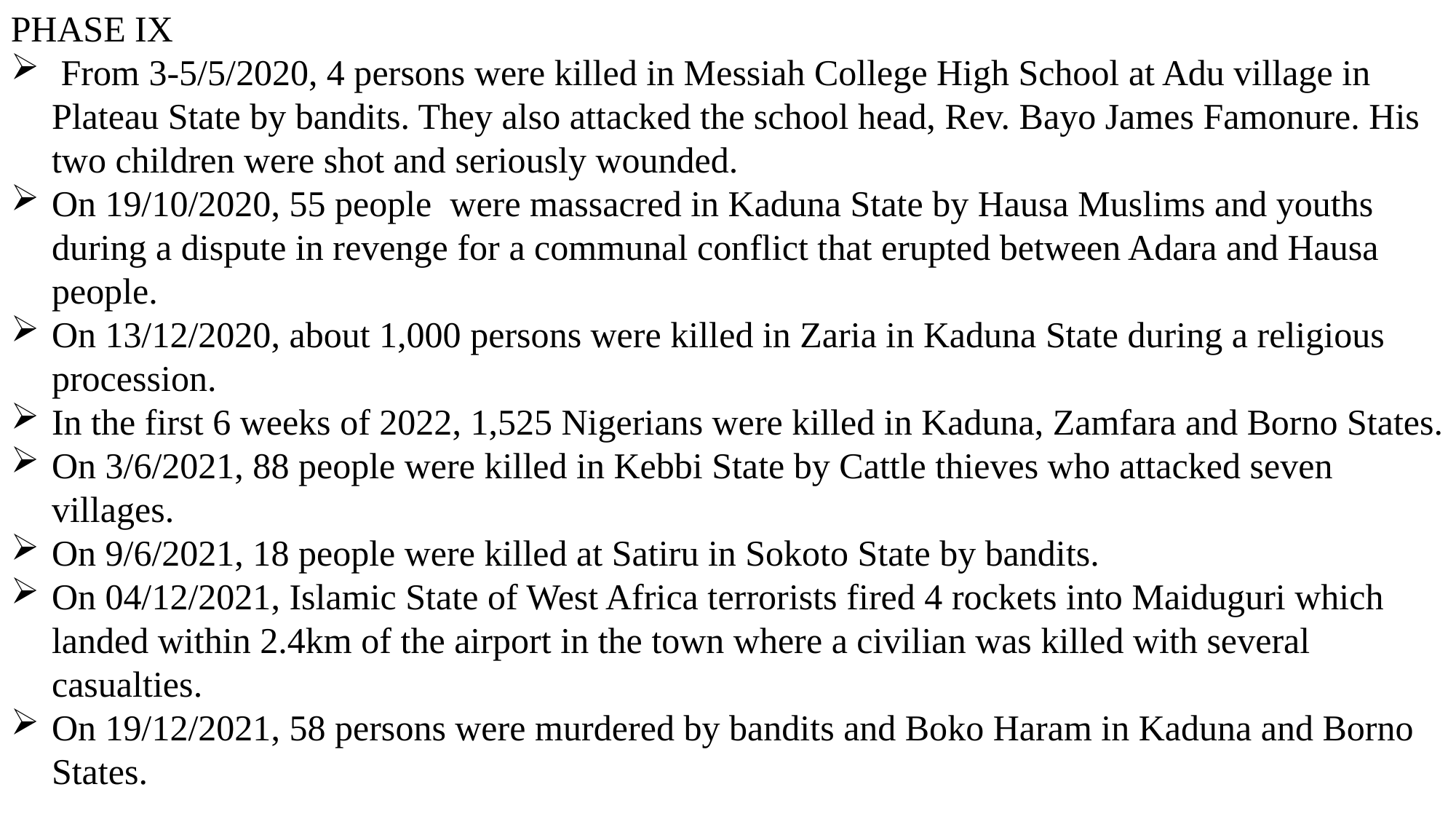

PHASE IX
 From 3-5/5/2020, 4 persons were killed in Messiah College High School at Adu village in Plateau State by bandits. They also attacked the school head, Rev. Bayo James Famonure. His two children were shot and seriously wounded.
On 19/10/2020, 55 people were massacred in Kaduna State by Hausa Muslims and youths during a dispute in revenge for a communal conflict that erupted between Adara and Hausa people.
On 13/12/2020, about 1,000 persons were killed in Zaria in Kaduna State during a religious procession.
In the first 6 weeks of 2022, 1,525 Nigerians were killed in Kaduna, Zamfara and Borno States.
On 3/6/2021, 88 people were killed in Kebbi State by Cattle thieves who attacked seven villages.
On 9/6/2021, 18 people were killed at Satiru in Sokoto State by bandits.
On 04/12/2021, Islamic State of West Africa terrorists fired 4 rockets into Maiduguri which landed within 2.4km of the airport in the town where a civilian was killed with several casualties.
On 19/12/2021, 58 persons were murdered by bandits and Boko Haram in Kaduna and Borno States.

## Slide 11
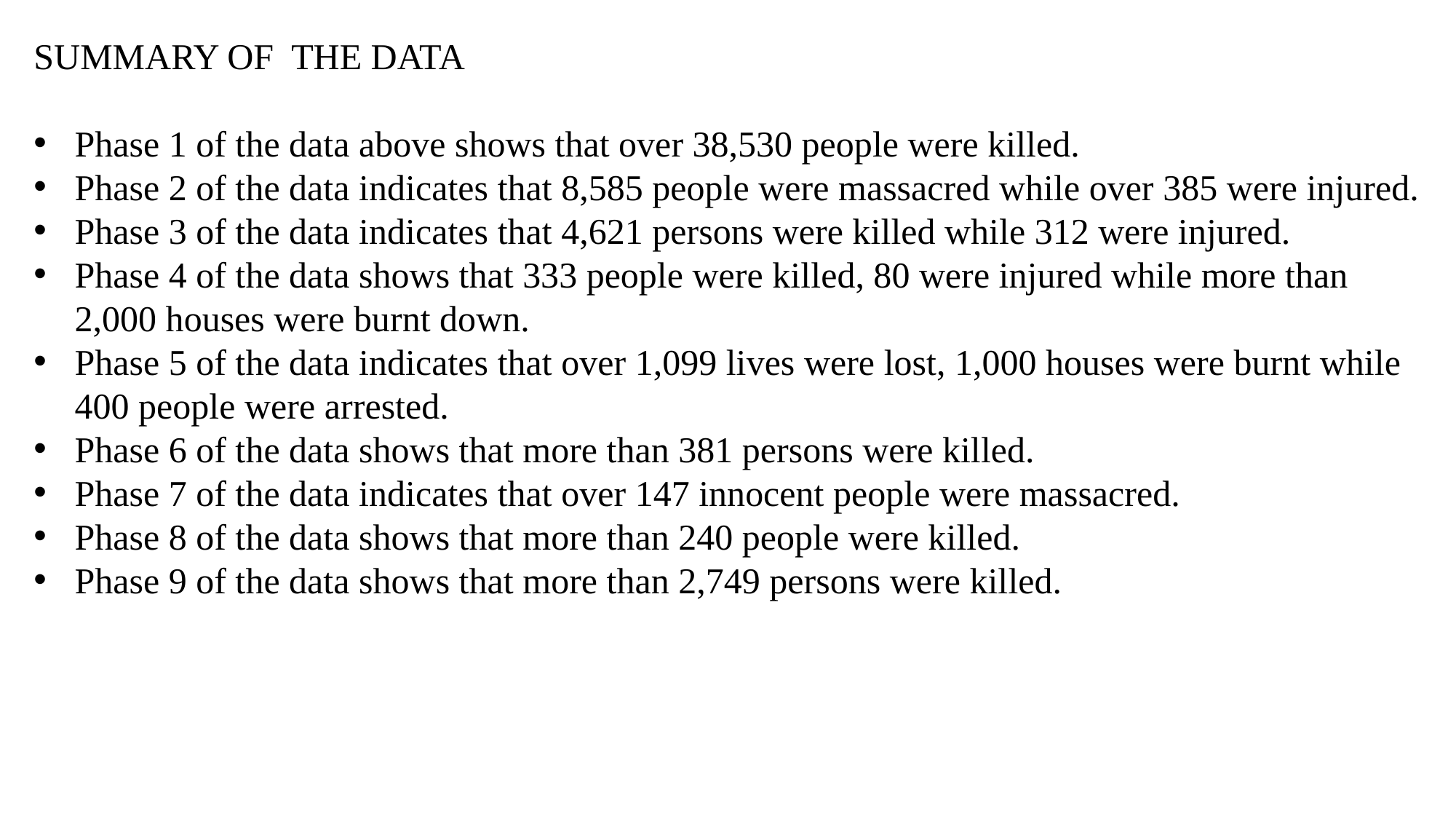

SUMMARY OF THE DATA
Phase 1 of the data above shows that over 38,530 people were killed.
Phase 2 of the data indicates that 8,585 people were massacred while over 385 were injured.
Phase 3 of the data indicates that 4,621 persons were killed while 312 were injured.
Phase 4 of the data shows that 333 people were killed, 80 were injured while more than 2,000 houses were burnt down.
Phase 5 of the data indicates that over 1,099 lives were lost, 1,000 houses were burnt while 400 people were arrested.
Phase 6 of the data shows that more than 381 persons were killed.
Phase 7 of the data indicates that over 147 innocent people were massacred.
Phase 8 of the data shows that more than 240 people were killed.
Phase 9 of the data shows that more than 2,749 persons were killed.

## Slide 12
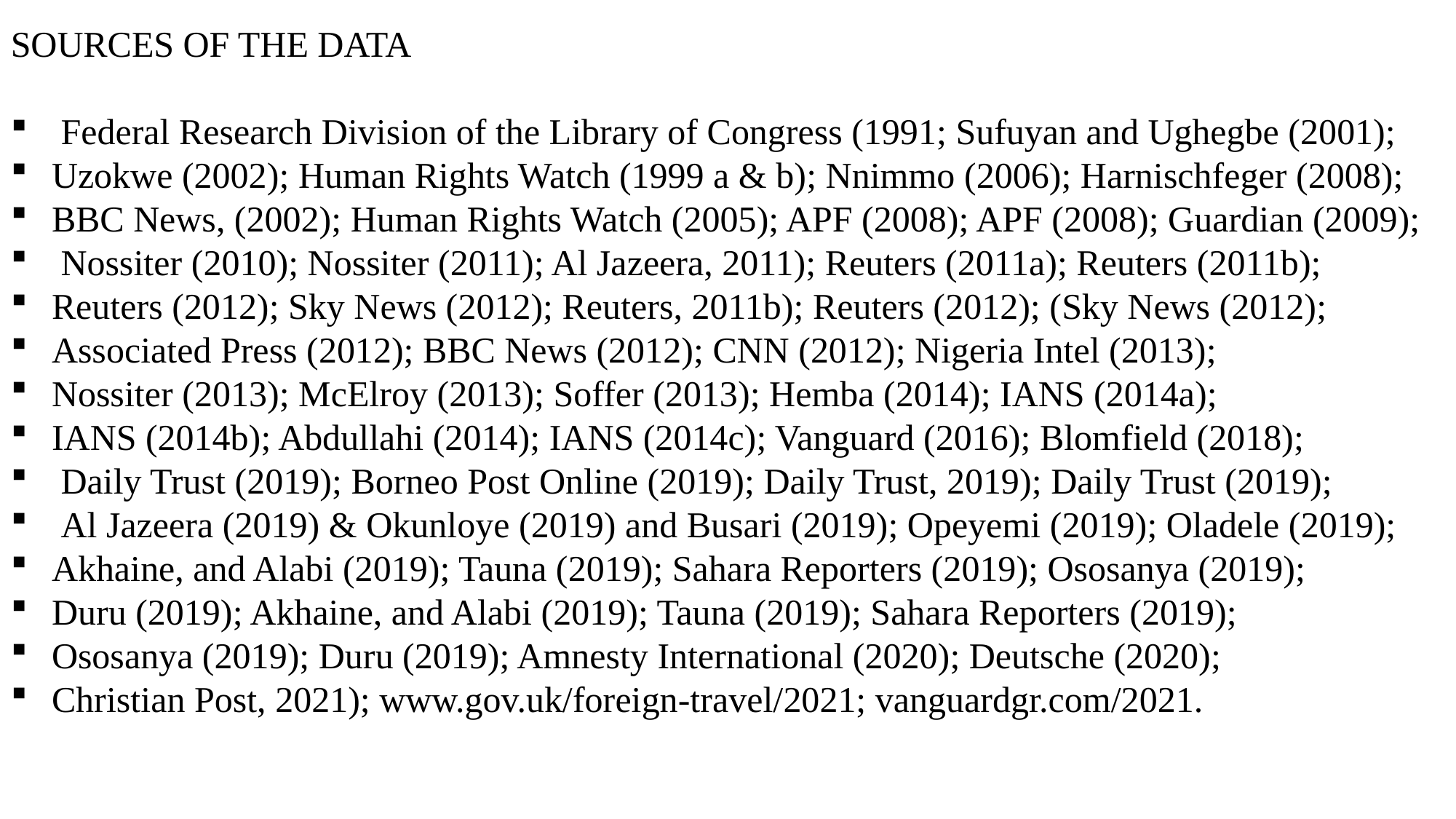

SOURCES OF THE DATA
 Federal Research Division of the Library of Congress (1991; Sufuyan and Ughegbe (2001);
Uzokwe (2002); Human Rights Watch (1999 a & b); Nnimmo (2006); Harnischfeger (2008);
BBC News, (2002); Human Rights Watch (2005); APF (2008); APF (2008); Guardian (2009);
 Nossiter (2010); Nossiter (2011); Al Jazeera, 2011); Reuters (2011a); Reuters (2011b);
Reuters (2012); Sky News (2012); Reuters, 2011b); Reuters (2012); (Sky News (2012);
Associated Press (2012); BBC News (2012); CNN (2012); Nigeria Intel (2013);
Nossiter (2013); McElroy (2013); Soffer (2013); Hemba (2014); IANS (2014a);
IANS (2014b); Abdullahi (2014); IANS (2014c); Vanguard (2016); Blomfield (2018);
 Daily Trust (2019); Borneo Post Online (2019); Daily Trust, 2019); Daily Trust (2019);
 Al Jazeera (2019) & Okunloye (2019) and Busari (2019); Opeyemi (2019); Oladele (2019);
Akhaine, and Alabi (2019); Tauna (2019); Sahara Reporters (2019); Ososanya (2019);
Duru (2019); Akhaine, and Alabi (2019); Tauna (2019); Sahara Reporters (2019);
Ososanya (2019); Duru (2019); Amnesty International (2020); Deutsche (2020);
Christian Post, 2021); www.gov.uk/foreign-travel/2021; vanguardgr.com/2021.
